# Non-operative Management and Outcomes of Femoroacetabular Impingement Syndrome

**DOI:** 10.1007/s12178-023-09863-x

**Published:** 2023-08-31

**Authors:** Rosa M. Pasculli, Elizabeth A. Callahan, James Wu, Niam Edralin, William A. Berrigan

**Affiliations:** 1 Department of Orthopaedics, Emory University School of Medicine, Atlanta, GA USA; 2 Department of Physical Medicine and Rehabilitation, New York University, New York, NY USA; 3 University of California Berkeley, Berkeley, CA USA; 4 Department of Orthopaedics, University of California San Francisco, 1500 Owens Street, San Francisco, CA 94158 USA

**Keywords:** Hip impingement, Femoroacetabular impingement syndrome, Conservative treatment, Physical therapy, Cam lesion, Pincer lesion

## Abstract

**Purpose:**

To serve as a guide for non-operative physicians in the management of femoroacetabular impingement syndrome and provide an algorithm as to when to refer patients for potential surgical management.

**Recent Findings:**

Supervised physical therapy programs that focus on active strengthening and core strengthening are more effective than unsupervised, passive, and non-core-focused programs. There is promising evidence for the use of intra-articular hyaluronic acid and PRP as adjunct treatment options. Recent systematic reviews and meta-analyses have found that in young active patients, hip arthroscopy demonstrates improved short-term outcomes over physical therapy.

**Summary:**

The decision for the management of FAIS is complex and should be specific to each patient. Consideration of the patient’s age, timing to return to sport, longevity of treatment, hip morphology, and degree of cartilage degeneration is required to make an informed decision in the treatment of these patients.

## Introduction

Femoroacetabular impingement (FAI) refers to abnormal early contact between the femoral head-neck junction and acetabular rim during hip joint functional range of motion. While FAI was first described in the literature in 1936, the definition underwent several iterations until a consensus statement was published in 2016 known as the Warwick Agreement [1–4]. The Warwick Agreement defined femoroacetabular impingement syndrome (FAIS) as a clinical triad of symptoms, signs, and imaging findings related to the underlying hip pathology [4].

**Fig. 1:**
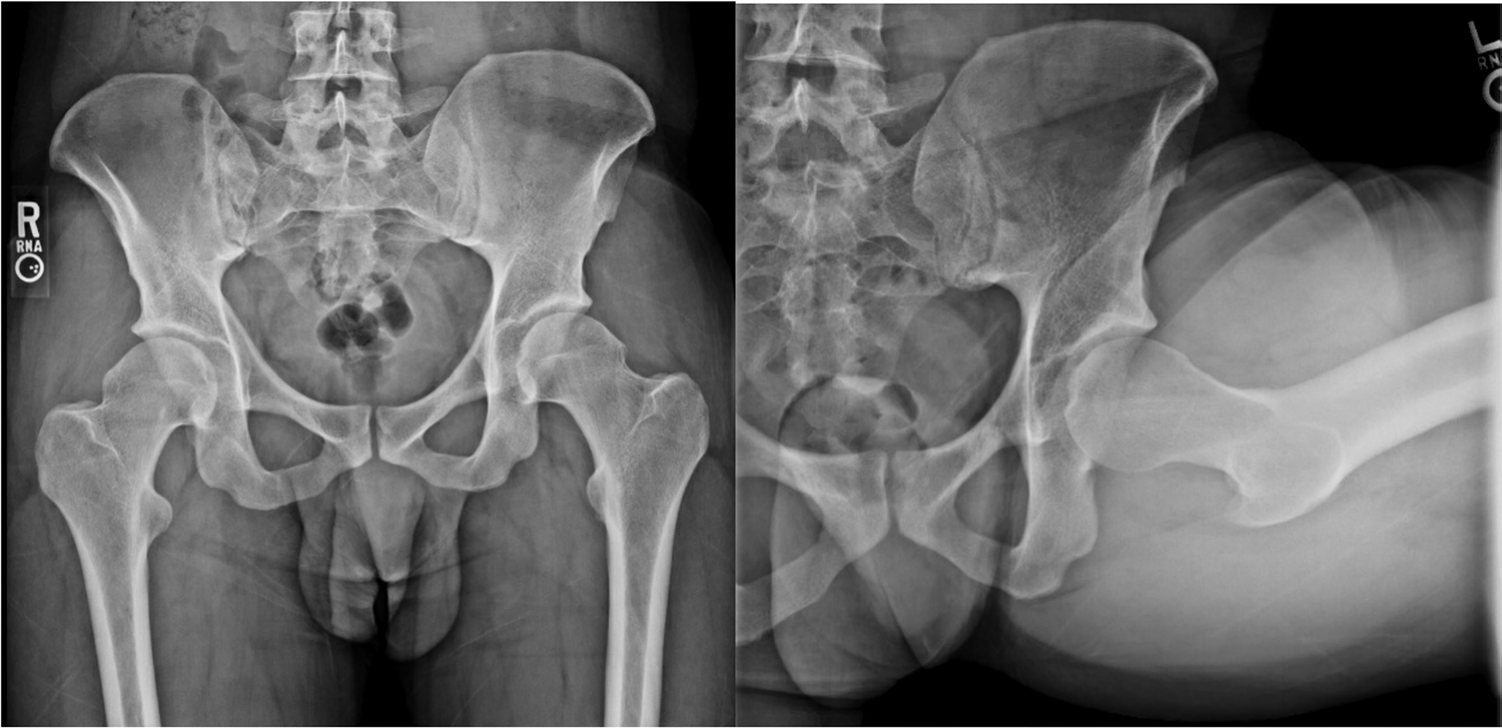
AP and Dunn lateral X-ray views demonstrating a classic cam deformity in a 29-year-old male with symptomatic FAIS. The alpha angle on this hip is 71° on the left with a Tonnis grade 1, Kellgren-Lawrence grade 1, and minimal osteophytic lipping

**Fig. 2:**
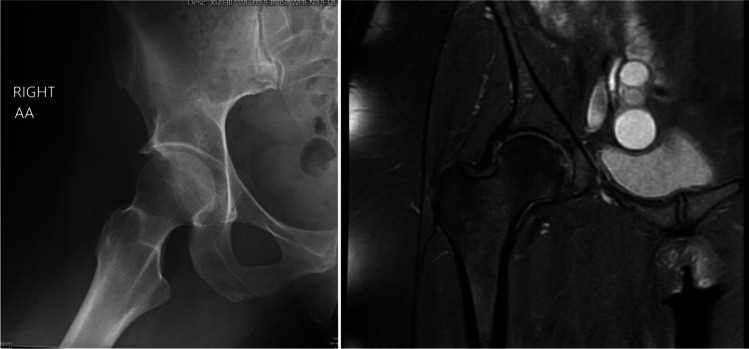
A) Frog leg view of the lateral hip in a 45-year-old female with symptomatic FAIS demonstrates a pincer lesion with Tonnis grade: 0–1 and alpha angle: 57. B) Corresponding coronal proton dense MRI of the right hip demonstrates labral hypertrophy with tearing and labral ossification resulting in the pincer lesion

There are three types of FAI morphologies that are defined by the underlying hip anatomy: cam, pincer, and mixed (Figs. 1 and 2). In the cam morphology, there is an aspherical femoral head resulting from bony overgrowth along the head-neck junction. Cam morphology represents approximately 37% of the population; it is more prevalent in males and in skeletally immature athletes who participate in high-intensity sports that require frequent hip loading with running, jumping, and kicking [5, 6]. It is believed that this repetitive injury to the proximal femoral physis experienced during adolescence can contribute to the development of a cam lesion [7]. As the hip is brought into flexion and internal rotation, shearing forces can cause damage to the acetabular cartilage and labrum along the anterosuperior joint [8]. In the pincer morphology, there is increased acetabular coverage of the femoral head, which can be associated with acetabular retroversion [9]. The prevalence of the pincer morphology is approximately 67% with similar rates seen in the general and athletic populations [10]. Acetabular over-coverage leads to labral damage as it gets impinged between the acetabular rim and femoral neck during flexion and may lead to intra-labral calcification or os acetabuli [11]. The mixed type morphology has both cam and pincer anatomic features and is more common than either cam or pincer alone [9].

The most reported symptoms in FAI impingement are hip or groin pain typically made worse with motion or in certain positions (i.e., prolonged sitting); however, patients may also report clicking, locking, or stiffness [4]. The most utilized clinical sign is the FADIR test (hip flexion, adduction, and internal rotation); however, it is neither sensitive nor specific for FAI impingement (estimated screening sensitivity 41% and specificity 47%) [12, 13]. Patients may also display a restricted hip range of motion, decreased hip strength, and impaired single-leg balance [14].

Initial diagnostic imaging of FAI syndrome should include 2 views of the affected hip: anteroposterior (AP) and lateral (either cross-table lateral or modified Dunn). There are several radiographic measures that have been described in the literature to evaluate the severity of FAI morphology; however, the Warwick Agreement did not define cutoff diagnostic values. The most used measurement to quantify cam morphology is the alpha angle. While the definition of cam pathology is controversial, a value of at least 50–60° has been proposed to increase the tool’s specificity [15]. The lateral center–edge angle is a measurement used in pincer morphologies; values > 40° indicate relatively increased superolateral acetabular coverage of the femoral head [16]. A positive crossover sign (also known as the figure of 8 sign) is a radiographic finding of acetabular retroversion and can also be seen with pincer morphology. Cross-sectional imaging such as magnetic resonance imaging (MRI), MR arthrogram, or CT should be considered to further assess the FAI morphology and to evaluate for chondral and/or labral lesions.

The Warwick Agreement stated that there is no high-level evidence for a definitive treatment algorithm for FAI syndrome [4]. Conservative treatment for FAIS consists of rest, patient education, activity and lifestyle modification, oral analgesia, physical therapy, and various intra-articular hip injections. Surgical intervention for FAIS involves hip arthroscopy to correct hip morphology to achieve impingement-free motion; this may consist of removing the impinging bone and/or addressing damage within the nearby articular cartilage and labrum. The purpose of this review is to discuss the literature that supports the various treatment measures and to suggest practice guidelines for how to approach non-operative treatment in these patients.

## Rehabilitation

All patients should undergo a trial of non-operative management prior to surgery, which has been shown to be successful in approximately 39–82% of FAIS cases [17]. These treatments focus on conservative care and physical therapist-led rehabilitation. Prospective and retrospective studies have shown that an initial trial with these non-surgical treatments for a minimum of 3 months can reduce pain and alleviate symptoms for up to 5 years; this suggests the potential to delay or avoid surgical care [18–21].

### Activity Modifications

The pain associated with repetitive bony impingement can considerably reduce patients’ hip range of motion (ROM). Repetitive hip flexion and internal rotation can also contribute to increased compressive forces in the anterior hip joint [22, 23]. These factors decrease daily functional activities and potentially increase the risk of cartilage injury and osteoarthritis [24]. Therefore, patient education and activity modification should be at the center of conservative care to avoid these positions of deep hip flexion and internal rotation [25]. It is recommended to avoid poor postural habits, static positioning, sitting cross-legged, and improper movement patterns such as full squat or hip adduction (pivoting on the affected side) [18, 26, 27]. Sports such as cycling, running on a treadmill, or running narrow straight trails should be avoided during initial treatment since they involve simultaneous hip flexion and internal rotation [25]. Running in a zigzag and wide course, which avoids excessive frontal and transverse plane hip motions, but requires some abduction and external rotation during turns, is a modification that can be made [18]. Walking and swimming can also benefit FAIS patients as a viable exercise alternative [25].

### Physical Therapy

Activity modifications and patient education should be accompanied by a rehabilitation program that addresses specific muscular imbalances. Multiple studies have demonstrated significant weakness of proximal hip musculature in patients with FAI syndrome compared to healthy controls, specifically the hip abductors, external rotators, flexors, extensors, and adductors [28–31]. A systematic review conducted by Freke et al. found the most common physical impairments associated with FAI syndrome are decreased hip muscle strength and impaired single-leg balance [14]. In a more recent study using cross-sectional imaging, certain hip muscles (gluteus maximus, gluteus minimus, and rectus femoris) were found to be atrophied on MRI as demonstrated by smaller cross-sectional area in patients with FAI syndrome compared to controls [32].

Correcting these deficits should be done through a supervised and progressive physical therapy program which has been shown to be more effective than a home exercise program alone [33–36]. The goals of physical therapy for FAI syndrome are to improve hip stability, neuromuscular control, strength, ROM, and movement patterns [4]. This usually involves several steps: assessing the patient’s pain, function, and hip ROM, prescribing an individualized and progressive manual therapy and exercise program, and educating the patient about their condition and its management [37].

Manual therapy is a common practice intended to alleviate pain and improve the quality and range of motion of the target joint and soft tissue structures in patients with hip disorders. Positive outcomes of manual therapy are primarily attributed to a neurophysiological response that allows for corresponding muscle relaxation and pain reduction without affecting bony abnormalities. Some forms of manual treatments include manual stretching, soft tissue mobilization, joint mobilization, and manipulation [35]. In the treatment of FAIS, hip joint mobilization and manipulation techniques (such as distraction, distraction with flexion, and anteroposterior glides) have been shown to relieve pain and improve ROM [37]. It is important to note that an increase in ROM should not be forced through end-of-range stretching into impingement positions, nor should exercise or movement into impingement positions be employed if the patient is symptomatic [38].

While manual therapy focuses on relaxing, lengthening, and repositioning hip muscles, exercise-based physical therapy focuses on muscle strengthening and functional training. As weakness in the hip abductors, external rotators, flexors, extensors, and adductors in patients with symptomatic FAI could worsen dynamic hip impingement, these muscles need to be addressed [39, 40]. Core stability is also integral to treatment success. A recent systematic review of the physical therapy treatment options included a meta-analysis of five randomized controlled trials and concluded that programs with active strengthening and core strengthening resulted in statistically significantly improved functional outcomes compared to programs that did not focus on core strengthening, were unsupervised, and consisted of passive modality-based treatment [36]. A wide variety of trunk stabilization exercises, such as bird dogs, planks, and side bridges, have been adopted in core muscle strength training to enhance the effect of hip muscle strength training and stabilize the lumbar vertebrae and pelvis [41].

### Outcomes with Physical Therapy

The success of non-operative management of FAIS has been established. Pennock et al. conducted a prospective study examining conservative treatment on 76 adolescents (93 hips) with FAIS [20]. All patients underwent non-operative treatment consisting of rest from their sport for 6 weeks, a mandatory well-defined physical therapy protocol, and activity modifications. At an average of 2-year follow-up, 70% responded to treatment, and another 12% responded to a steroid injection, with an 82% overall success rate of avoiding surgery. As part of the same working group, Zogby et al. demonstrated that this effect of conservative treatment could be successful for up to 5 years [21]. It is worthwhile to note that in the original study, patients with larger cam and mixed cam/pincer deformities were four times more likely to progress to surgery (*p* = 0.05) than patients with pincer impingement. The majority of RCTs and pilot trials has examined the effectiveness of various physical therapy programs and has demonstrated improved short-term outcomes in hip pain and function, but only up to 2 years maximum post-intervention [41–43]. To our knowledge, only one other trial has examined mid-term outcomes after conservative treatment; while this was a small study, they did conclude a significant improvement in hip pain, function, and quality of life after completing a 12-week physical therapy program which persisted for an average of 4.6 years [44].

## Intra-articular Hip Injections

Intra-articular injections have been used to aid in the diagnosis of FAIS by differentiating intra-articular versus extra-articular etiologies of hip pain [45]. However, more recent data has looked at the therapeutic benefits (Table 1). Many reports and a few studies have demonstrated the efficacy of corticosteroids and hyaluronic acid (HA) intra-articular injections in patients with symptomatic FAI. A small, open prospective trial demonstrated that two intra-articular injections of moderate molecular weight hyaluronic acid (Sinovial Forte, 1200 kDa) 40 days apart with repeat injection at 6 months are a well-tolerated therapy for FAI which may provide long-lasting pain relief and functional improvement at 12 months [46]. This study was included in a systematic review of intra-articular injections for FAIS, which found that hyaluronic acid intra-articular injections were most effective in providing pain relief with significant improvements in functional outcome scores at 12 months, compared to pooled results with corticosteroid injections resulting in improvement of only 15% of patients at 6 weeks [47].

**Table 1:** Intra-articular injections for the treatment of FAIS

Author	Primary intervention	Comparator	Study design	N	Outcome measure	Follow-up (months)	Results	AEs	Conclusion
Abate et al. 2014	2 HA injections spaced out 40 days (0,6 mos)	None	Prospective cohort	23	Pain score, LISOH, mHHS, NSAID use	6, 12	Pain decreased at 6 and 12 months; LISOH decreased, mHHS improved at 12-month NSAID consumption reduced	Two local side effects	HA is safe and effective in the treatment of mild FAI, with significant pain reduction and functional improvement
Park et al. 2013	IA triamcinolone	None	Case series	3	VAS, OHS	0.5, 1, 2, 3	OHS scores for all patients decreased at 3 months	None	IA steroid reduced the pain and improved function in 3 cases of FAI
Krych et al. 2014	IA methyl-prednisolone acetate, triamcinolone, or betamethasone	None	Case series	54	NRS	0.5, 1.5	NRS decreased from post-injection anesthetic phase to 14 days. At 14 days, 20 patients, and at 6 weeks, 3 patients had clinically significant pain reduction	ND	Six percent of patients had sustained pain relief at 6 weeks. IA CSI has limited clinical benefit as a therapeutic modality
Lee et al. 2016	HA injections (Hyruan plus Inj 2 ml/syringe)	IA CSI (triamcinolone)	Randomized crossover trial	30	NRS, HOOS, Oral medication	3	Seventeen patients w/o crossover, HOOS at 2 weeks improved w/ HA injection. Thirteen patients w/ crossover, NRS improved at 2 weeks w/ 1st TA injection w/o difference in HOOS. At 4 weeks, decrease of NRS was greater w/ 1st HA and 2nd TA injections; HOOS improved greater in 1st HA and 2nd TA injections	8-facial flushing, menstrual irregularity, local site, effects	IA CSI had faster effect in pain relief. IA HA group had delayed effect in functional improvement
Pennock et al. 2018	Initial trial of rest, PT, activity modification followed by IA triamcinolone, followed by arthroscopic surgery	None	Prospective cohort	93	Return to sport, mHHS, NAHS	12,24	Seventy percent responded to conservative measures 12% required CSI; 18% required surgery. All groups saw similar improvements in mHHS and NAHS. Cam and cam-pincer were 4 × more likely to require surgical intervention	ND	Eighty-two percent of adolescent patients with FAI can be managed non-operatively, with significant improvements in outcome scores at a mean follow-up of 2 years
Ebert et al. 2023	IA triamcinolone w/ 12-week rehabilitation program	None	Case series	44	iHOT, HOS, mHHS, Tegner, VAS-S, ROM, Isometric strength	4, 6, 12, 24	31.8% of patients progressed to surgical intervention over the 24-month post-injection period. In the non-operative cohort, a significant improvement in all patient-reported outcome measures were observed, with 93% satisfaction	None	Although 32% of patients progressed to surgery, significant improvement in hip pain, symptoms, and physical function was observed in patients because of a targeted non-operative management
Hunt et al. 2012	Diagnostic IA triamcinolone + lidocaine	None	Prospective observational outcomes study	52	NPRS, SF-12 mHHS, WOMAC NAHS, Baecke Questionnaire of Habitual Activity	3, 12	Twenty-three (44%) of patients reported satisfaction with conservative care. 56% chose to have surgery. Both groups demonstrated equally significant improvement in all outcome measures from baseline to 1 year	ND	A trial of conservative management for persons with prearthritic, intra-articular hip disorders should be considered before engaging in surgical intervention
De Luigi et al. 2019	IA LR-PRP	None	Prospective cohort	8	VAS, HHS	0.5, 1.5, 2	Statistically significant improvements in VAS were seen with both rest and activity at 2 weeks, 6 weeks, and 8 weeks after PRP injection	None	Ultrasound-guided PRP injections hold promise as an emerging technique toward symptom relief, reducing pain, and improving function in patients with hip labral tears

*HA* hyaluronic acid, *mos* months, *IA* intra-articular, *mL* milliliter, *LR* leukocyte rich, *PRP* platelet-rich plasma, *CSI* corticosteroid injection, *LISOH* Lequesne Index for Severity of Osteoarthritis of the Hip, *mHHS* modified Hip Harris Score, *NSAID* nonsteroidal anti-inflammatory, *VAS* Visual Analog Scale, *OHS* Oxford Hip Score, *NRS* Numerical Rating Scale, *HOOS* Hip Disabilities and Osteoarthritis Outcome Score, *NAHS* Non-arthritic Hip Score, *iHOT* International Hip Outcome Tool, *HOS* Hip Outcome Score, *ROM* range of motion, *NPRS* Numerical Pain Rating Scale, *SF-12* 12 Item Short Form Survey, *WOMAC* Western Ontario and McMaster Universities Osteoarthritis Index

In addition, a randomized double-blind crossover study by Lee et al. examining intra-articular hip corticosteroid versus HA injections in FAIS found that corticosteroids resulted in faster pain relief at 2 weeks; however, patients who received hyaluronic acid had a more delayed functional improvement [48]. It is hypothesized that the clinical efficacy of hyaluronic acid in FAIS may be due to its mechanical effects, along with its protective properties on articular cartilage; it acts as a lubricant, improves the viscoelasticity of the joint, contributes to increased synovial fluid, and has anti-inflammatory and anti-nociceptive effects [46].

The effect of corticosteroids in patients with symptomatic FAI is less clear. Krych et al. evaluated the efficacy of a single intra-articular hip corticosteroid injection in patients with FAIS and/or labral tears and found a limited benefit of intra-articular corticosteroid injection in patients with FAIS, with only 37% of patients reporting significantly diminished pain at 2 weeks, and only 6% of patients reporting sustained relief at 6 weeks (average duration of pain relief was 9.8 days) [49]. A case series of three patients did show significantly reduced pain and improved function sustained at 3 months after an intra-articular hip corticosteroid injection, although this was a small sample size [50]. More recently, a larger case series by Ebert et al. examined 44 patients with FAIS and found significant improvement in pain and function after intra-articular corticosteroid injection followed by a structured 12-week rehabilitation program. While these improvements were largely sustained at 24 months, it should be noted that 32% of patients progressed to surgery [51]. It is important to note that these studies include a heterogeneous population which makes it difficult to draw firm conclusions regarding the benefit of corticosteroid injections in FAIS. It can be suggested that age and symptom severity likely influence which patients will benefit from intra-articular corticosteroid and hyaluronic acid hip injections.

Lastly, the use of platelet-rich plasma (PRP) intra-articular hip injections seems to hold promise as a non-operative treatment for hip labral tears [52–54]. De Luigi et al. found that leukocyte-rich PRP use is a potentially safe treatment option for hip labral tears and improved Harris Hip Scores and Visual Analog Scale scores at 2 weeks, 6 weeks, and 8 weeks post-injection [54]. This study only included patients with labral tearing identified on MRI, positive hip impingement testing, and without severe osteoarthritis. While FAIS was not directly studied in this prospective cohort, we know that FAIS is commonly seen concomitantly with a hip labral tear, and therefore, it can be hypothesized that PRP could be a safe, promising treatment option for symptomatic FAI. Long-term studies in this area are needed, and future research is warranted. However, given the multiple systematic reviews and meta-analyses demonstrating the superiority of PRP to hyaluronic acid in the treatment of knee osteoarthritis, PRP could be considered for patients with symptomatic FAIS as an alternative to corticosteroid and hyaluronic acid [55–57].

It should be noted that these observational studies have notable limitations, including relatively small sample sizes, heterogeneous study populations, and open study design lacking a control arm. Future trials should continue to examine the efficacy of various intra-articular hip injections as another potential conservative treatment for FAIS.

## Conservative Management Versus Hip Arthroscopy

There exist many systematic reviews and meta-analyses that include multiple level one randomized clinical trials comparing physical therapy to hip arthroscopy for FAIS. The consensus based on the most recent reviews is that surgery demonstrates improved short-term outcomes (< 1 year) over physical therapy in young and active patients; however, the authors acknowledge that physical therapy can also have a positive effect [58, 59].

The conclusions of these reviews are based on four major clinical trials. Griffin et al. in the UK FASHIoN study conducted a multicenter randomized trial of 348 patients (average age of 35) that were allocated to receive hip arthroscopy or personalized hip physical therapy program for 12–24 weeks [60]. Both groups demonstrated improvement in patient-reported outcomes, but those treated with hip arthroscopy had a higher statistically significant improvement in symptoms with decreased functional limitations when compared with the conservative arm at 12 months. Of note, this trial did not include injections as an additional non-surgical intervention. In a qualitative follow-up study of 40 study patients, both arms felt the intervention was beneficial, but treatment success appeared to depend partly on the patients’ prior own expectations in addition to their outcomes [61].

Palmer et al. had similar findings in the FAIT study, a multicenter RCT of 222 participants (average age of 36) which compared eight sessions of physical therapy to arthroscopic surgery. At 8 months post-intervention, the surgical group had significantly improved functional outcomes (measured by the hip outcome score activities of daily living) compared to the non-surgical group [62]. Hunter et al. in the Australian FASHIoN multicenter trial of 99 participants (average age of 33) found a significant improvement in both the physical therapy and arthroscopy groups, but the arthroscopy group demonstrated a significant difference in clinical improvement over physical therapy at 1 year [63].

These three published RCTs found small to moderate between-group differences favoring hip arthroscopy. Only one study found contrary results. A smaller RCT of 80 patients in the military population by Mansell et al. randomized patients to either hip arthroscopy or 12 sessions of physical therapy. At 2 years, there were no significant functional differences between groups. Interestingly, 33% of patients were unable to return to active military duty regardless of treatment [64]. The lower return rate to active-duty military may be due to multiple factors including a higher level of activity than average patients amongst other studies. It should be noted that this study included a higher rate of crossover from non-operative management to surgery (70%) than the previous studies.

There are several limitations throughout these studies. Certain physical therapy and exercise protocols that served as control interventions in previous RCTs may not represent the current best practice for conservative management of FAIS [65]. In some RCTs, patient recruiting criteria were not well-defined, and some patients may have had labral tears, cartilage damage, and/or limited radiographic osteoarthritis before treatment (although trials did not include patients with Tonnis > 1 or KL > 2), which may confound the clinical picture and results. Furthermore, most RCTs only evaluated short-term outcomes (≤ 2 years), leaving the mid- and long-term benefits of both hip arthroscopic surgery and physical therapy to be investigated [21, 44]. Further high-powered longitudinal studies are needed.

## Referral for Hip Arthroscopy Consultation

### Osteoarthritis

Hip arthroscopy is generally contraindicated in the setting of moderate to advanced hip osteoarthritis. Many prospective studies demonstrate osteoarthritis as a negative predictor of outcomes. A systematic review by Domb et al. looked at 2051 hips that underwent arthroscopy and found that patients with a Tonnis grade of 1 or greater, or a joint space of 2 mm or less, were less likely to benefit from hip arthroscopy and more likely to require conversion to total hip arthroplasty [66]. A separate review by Kemp et al. showed a mean time of progression to total hip replacement to be between 7 months and 4.8 years in patients that had recognized arthritis [67].

Evidence is currently lacking to support the use of initial arthroscopy for FAIS to prevent later development of hip OA. A study by Rhon et al. done in the military population of 1870 participants (mean age of 32) found a clinical diagnosis of hip osteoarthritis in 22% of the population within 2 years of arthroscopic surgery for FAIS [68]. Collins et al. queried whether prophylactic surgery was indicated for FAI and concluded that the evidence does not support prophylactic surgery for FAI in most cases, but rather may result in 80% of patients with asymptomatic FAI undergoing an unnecessary procedure [69]. Undergoing surgery, according to the Australian FASHIoN RCT, may also have less of a cartilage preservation effect than a non-surgical treatment option [63].

### Morphological Factors

There is very limited data suggesting that physical therapy may be less successful with more severe cam deformities. In a small study of 31 patients, Casartelli et al. showed severe cam deformities to be present in 40% of patients that did not respond to a standard exercise therapy program versus only being present in 6% that had improved with treatment [42]. Morphological factors have been further studied in the arthroscopy literature, but this is beyond the scope of this review [70, 71].

Cam-type FAIS has been associated with the radiographic development of hip osteoarthritis. In a population-based 20-year longitudinal cohort study of 1003 women, each degree that the alpha angle increased beyond 65° increased the odds ratio by 5% for hip OA development and increased the risk of total hip replacement by 4% [72]. The Cohort Hip and Cohort Knee (CHECK) study made similar conclusions. The CHECK group found that a baseline A P alpha angle > 83° had a 25% risk of developing end-stage OA within 5 years compared with a < 2% risk of end-stage OA in hips with an alpha angle of < 83°. Hips with both an alpha angle > 83° and decreased internal rotation ≤ 20° had a risk of 53% for end-stage OA within 5 years [73].

### Patient Age

When considering conservative management or surgical referral for a patient with FAIS, the patient’s age is one of the most important demographic factors to contemplate. As previously described above, Pennock et al. demonstrated the success of non-operative management in adolescents with FAIS; 82% of symptomatic hips significantly improved in functional outcome scores at 2 years with rest, activity modification, physical therapy, and intra-articular steroid injections [20]. This clinical improvement persisted at 5 years post-intervention [21]. For skeletally immature patients, there are unique, theoretical surgical risks to this population, such as osteonecrosis and epiphyseal injury. While there is limited data on surgical outcomes after arthroscopy in skeletally immature patients with FAIS, a recent systematic review investigating the role of arthroscopic management in adolescents with FAIS showed significantly improved pain and functional outcomes at a mean follow-up of 30.4 months with low rates of complications and revision surgeries [74]. Another recent observational study by Fukase et al. demonstrated a physeal-sparing hip arthroscopy in adolescents with FAIS was safe with significantly improved pain and functional outcomes at 8.9 years and also had significantly improved patient-reported outcomes compared to matched adults at 6.6 years [75]. For the physeal-sparing surgery, the impingement was preferably addressed on the acetabular side, and a limited approach was taken with the cam resection to avoid damaging the growth plate. If there was a significant cam lesion in contact with the physis, only the acetabular osteoplasty and labral treatment were addressed in the surgery.

A recent cohort study by Lin et al. examined the outcomes of hip arthroscopy in FAIS in patients stratified into three age groups: young (mean 27.7 years), middle (mean 41.5 years), and old (mean 60.2 years) [76]. While all three groups had significantly improved patient-reported outcomes at 5 years, the middle-aged and older patients experienced greater declines in clinical outcomes over time than younger patients, and the older patients had a higher risk of progression to total hip arthroplasty (THA). A prospective comparative matched group analysis by Frank et al. demonstrated that patients > 45 years old had significantly worse hip outcome scores 2 years after hip arthroscopy for FAIS compared with cohorts 30–45 years old and < 30 years old [77]. Similar findings were demonstrated in a larger case control study examining outcomes after hip arthroscopy for FAIS where patients < 30 years old achieved patient acceptable symptomatic state (PASS) on hip outcome and pain scores at higher rates than patients > 45 years old [78]. A 2014 systematic review also showed that older age was associated with worse postoperative outcomes and increased risk of subsequent THA, although a specific age cutoff was not defined [79]. For older patients, there may be limited indications for hip arthroscopy given the high prevalence of preexisting hip OA and degenerative labral tears in this population and worse outcomes when compared to younger cohorts. The rate of conversion to THA after hip arthroscopy increases with each decade, starting at approximately 18.1% for patients 40 or older, 23.1% for patients over 50, and 25.2% for patients over 60 with a mean of 25.0 months to THA [80].

For young to middle-age active patients with FAIS, we recommend initiating conservative treatment for at least 3 months; however, after that point, they may benefit from an earlier surgical referral than older patients. For older patients, we do not see a role for hip arthroscopy; instead, they may benefit from a referral for THA after failure of conservative treatment.

### Timing of Arthroscopy

Expert consensus opinion is that patients with FAIS whose symptoms and function fail to improve after 3–6 months of conservative treatment should be referred to an orthopedic surgeon to discuss arthroscopic intervention [81]. We have questioned if there is ideal timing of surgical intervention and if symptom duration of FAIS affects outcomes after surgery. This was examined in a recent cohort study by Kunze et al. (*N* = 1049, mean age of 32.3 years) who demonstrated that FAIS patients who underwent hip arthroscopy after 3–6 months of symptoms had significantly improved postoperative pain scores and superior International Hip Outcome-12 scores compared to those patients with 6–12 months, 12–24 months, and > 24 months of symptom duration before hip arthroscopy [82]. A systematic review by Saadat et al. showed that increased duration of FAIS symptoms of > 1.5 years was associated with worse postoperative outcomes and increased risk of subsequent THA [79]. It should be noted that these improved postoperative outcomes associated with shorter duration of symptoms are similar to those seen in FAIS patients treated conservatively; Monn et al. reported that patients with a low pain level and short duration of FAI symptoms had improved mid-term outcomes at 4.6 years after an initial 12-week exercise therapy program [44].

## Return to Play

Both athletes and patients who are physically active with FAIS may be concerned about time to return to play and performance level in their sport. There is a lack of evidence about return-to-play outcomes after conservative management, with more robust data after hip arthroscopy. A recent systematic review examined return to play after hip arthroscopy in FAIS and found an overall return to play rate of 85.4% over an average of 6.6 months [83]. While the overall quality of evidence was low, an important takeaway from this article is that ¼ of athletes did not return at their preinjury level. This finding was demonstrated in another systematic review and meta-analysis which showed that one in four athletes did not return to their previous level of sport participation after arthroscopy treatment for FAIS [84]. Another systematic review and meta-analysis assessed outcomes after hip arthroscopy in FAIS and found an 87.7% rate of return to sport [85]. Many of the studies included in these reviews advised return to play beginning 3–6 months after hip arthroscopy, while some suggested a more conservative timeline up to 10 months. Return to play for any athlete should be guided by a patient-centered discussion with focused rehabilitation that includes sport-specific goals and demands.

## Conclusion

Although hip arthroscopic surgery for FAIS is increasing in prevalence, the benefits of surgery compared with non-operative treatment still require more research and consideration. Conservative care is commonly accepted as the first step in treatment given a moderate chance of improvement with cost efficiency and a low risk of harm. Detailed and standardized physical therapy protocols and measurement methods still need to be developed to maximize results.

In those where the diagnosis is unclear, or in FAIS patients that have not responded to physical therapy, but are not ready for surgical intervention, an intra-articular hip injection is a reasonable next step. There are a small number of observational studies looking at the efficacy of intra-articular hip injections as an adjunct treatment for FAIS. These studies include relatively small sample sizes, heterogeneous study populations, and open study design lacking a control arm. Future trials are needed to determine the efficacy of various injections, including the long-term safety and efficacy of hyaluronic acid and PRP injections, which show promising initial evidence.

**Fig. 3:**
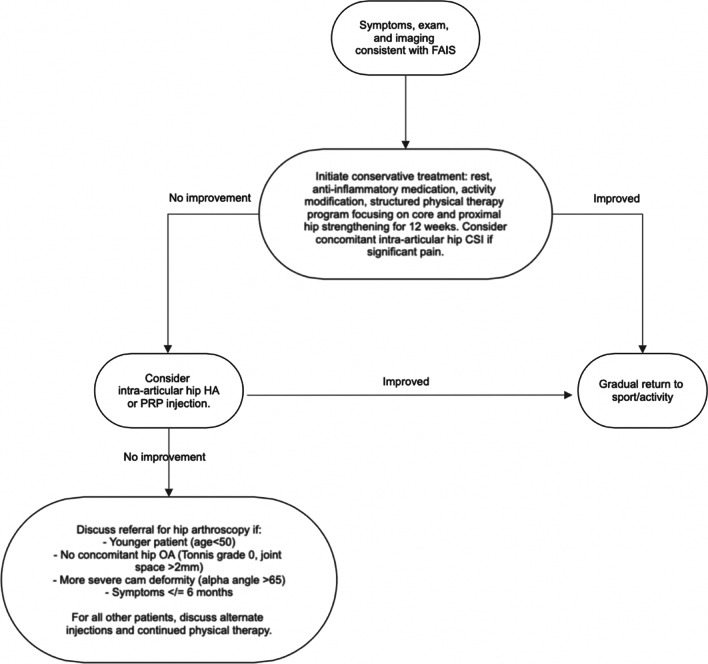
Recommendations for the clinician’s approach to the management of FAIS

The algorithm for how to manage FAIS is complex and should be specific to each patient. Consideration of the patient’s age, timing to return to sport, longevity of treatment, hip morphology, and degree of cartilage degeneration is required to make an informed decision in the treatment of these patients. We have summarized our recommendation for the general approach and treatment of FAIS in Fig. 3.

## References

[1] Smith-Petersen MN (2009). The classic: treatment of malum coxae senilis, old slipped upper femoral epiphysis, intrapelvic protrusion of the acetabulum, and coxa plana by means of acetabuloplasty. 1936. Clin Orthop Relat Res..

[2] Ganz R, Gill TJ, Gautier E, Ganz K, Krügel N, Berlemann U (2001). Surgical dislocation of the adult hip a technique with full access to the femoral head and acetabulum without the risk of avascular necrosis. J Bone Joint Surg Br.

[3] Sankar WN, Nevitt M, Parvizi J, Felson DT, Agricola R, Leunig M (2013). Femoroacetabular impingement: defining the condition and its role in the pathophysiology of osteoarthritis. J Am Acad Orthop Surg.

[4] Griffin DR, Dickenson EJ, O'Donnell J, Agricola R, Awan T, Beck M (2016). The Warwick Agreement on femoroacetabular impingement syndrome (FAI syndrome): an international consensus statement. Br J Sports Med.

[5] Frank JM, Harris JD, Erickson BJ, Slikker W, Bush-Joseph CA, Salata MJ (2015). Prevalence of femoroacetabular impingement imaging findings in asymptomatic volunteers: a systematic review. Arthroscopy.

[6] Packer JD, Safran MR (2015). The etiology of primary femoroacetabular impingement: genetics or acquired deformity?. J Hip Preserv Surg.

[7] Ezechieli M, Banke IJ (2022). Epidemiology, prevention and early detection of femoroacetabular impingement syndrome (FAIS). Orthopade.

[8] Beck M, Kalhor M, Leunig M, Ganz R (2005). Hip morphology influences the pattern of damage to the acetabular cartilage: femoroacetabular impingement as a cause of early osteoarthritis of the hip. J Bone Joint Surg Br.

[9] Trigg SD, Schroeder JD, Hulsopple C (2020). Femoroacetabular Impingement Syndrome. Curr Sports Med Rep.

[10] Mascarenhas VV, Rego P, Dantas P, Morais F, McWilliams J, Collado D (2016). Imaging prevalence of femoroacetabular impingement in symptomatic patients, athletes, and asymptomatic individuals: a systematic review. Eur J Radiol.

[11] Larson CM, LaPrade RF, Floyd ER, McGaver RS, Bedi A (2021). Acetabular rim disorders/pincer-type femoroacetabular impingement and hip arthroscopy. Sports Med Arthrosc Rev.

[12] Reiman MP, Goode AP, Cook CE, Hölmich P, Thorborg K (2015). Diagnostic accuracy of clinical tests for the diagnosis of hip femoroacetabular impingement/labral tear: a systematic review with meta-analysis. Br J Sports Med.

[13] Casartelli NC, Brunner R, Maffiuletti NA, Bizzini M, Leunig M, Pfirrmann CW (2018). The FADIR test accuracy for screening cam and pincer morphology in youth ice hockey players. J Sci Med Sport.

[14] Freke MD, Kemp J, Svege I, Risberg MA, Semciw A, Crossley KM (2016). Physical impairments in symptomatic femoroacetabular impingement: a systematic review of the evidence. Br J Sports Med.

[15] Sutter R, Dietrich TJ, Zingg PO, Pfirrmann CW (2012). How useful is the alpha angle for discriminating between symptomatic patients with cam-type femoroacetabular impingement and asymptomatic volunteers?. Radiology.

[16] Welton KL, Jesse MK, Kraeutler MJ, Garabekyan T, Mei-Dan O (2018). The anteroposterior pelvic radiograph: acetabular and femoral measurements and relation to hip pathologies. J Bone Joint Surg Am.

[17] Schwabe MT, Clohisy JC, Cheng AL, Pascual-Garrido C, Harris-Hayes M, Hunt DM (2020). Short-term clinical outcomes of hip arthroscopy versus physical therapy in patients with femoroacetabular impingement: a systematic review and meta-analysis of randomized controlled trials. Orthop J Sports Med.

[18] Emara K, Samir W, Motasem EH, Ghafar KA (2011). Conservative treatment for mild femoroacetabular impingement. J Orthop Surg (Hong Kong)..

[19] Kekatpure AL, Ahn T, Kim CH, Lee SJ, Yoon KS, Yoon PW (2017). Clinical outcomes of an initial 3-month trial of conservative treatment for femoroacetabular impingement. Indian J Orthop.

[20] Pennock AT, Bomar JD, Johnson KP, Randich K, Upasani VV (2018). Nonoperative management of femoroacetabular impingement: a prospective study. Am J Sports Med.

[21] Zogby AM, Bomar JD, Johnson KP, Upasani VV, Pennock AT (2021). Nonoperative management of femoroacetabular impingement in adolescents: clinical outcomes at a mean of 5 years: a prospective study. Am J Sports Med.

[22] Jorge JP, Simões FM, Pires EB, Rego PA, Tavares DG, Lopes DS (2014). Finite element simulations of a hip joint with femoroacetabular impingement. Comput Methods Biomech Biomed Engin.

[23] Bagwell JJ, Powers CM (2017). The influence of squat kinematics and cam morphology on acetabular stress. Arthroscopy.

[24] Agricola R, Waarsing JH, Arden NK, Carr AJ, Bierma-Zeinstra SM, Thomas GE (2013). Cam impingement of the hip: a risk factor for hip osteoarthritis. Nat Rev Rheumatol.

[25] Loudon JK, Reiman MP (2014). Conservative management of femoroacetabular impingement (FAI) in the long distance runner. Phys Ther Sport.

[26] Lamontagne M, Kennedy MJ, Beaulé PE (2009). The effect of cam FAI on hip and pelvic motion during maximum squat. Clin Orthop Relat Res.

[27] Terrell SL, Olson GE, Lynch J (2021). Therapeutic exercise approaches to nonoperative and postoperative management of femoroacetabular impingement syndrome. J Athl Train.

[28] Casartelli NC, Maffiuletti NA, Item-Glatthorn JF, Staehli S, Bizzini M, Impellizzeri FM (2011). Hip muscle weakness in patients with symptomatic femoroacetabular impingement. Osteoarthritis Cartilage.

[29] Nepple JJ, Goljan P, Briggs KK, Garvey SE, Ryan M, Philippon MJ (2015). Hip strength deficits in patients with symptomatic femoroacetabular impingement and labral tears. Arthroscopy.

[30] Kierkegaard S, Mechlenburg I, Lund B, Søballe K, Dalgas U (2017). Impaired hip muscle strength in patients with femoroacetabular impingement syndrome. J Sci Med Sport.

[31] Rutherford DJ, Moreside J, Wong I (2018). Differences in hip joint biomechanics and muscle activation in individuals with femoroacetabular impingement compared with healthy, asymptomatic individuals: is level-ground gait analysis enough?. Orthop J Sports Med.

[32] Malloy P, Stone AV, Kunze KN, Neal WH, Beck EC, Nho SJ (2019). Patients with unilateral femoroacetabular impingement syndrome have asymmetrical hip muscle cross-sectional area and compensatory muscle changes associated with preoperative pain level. Arthroscopy.

[33] Harris-Hayes M, Czuppon S, Van Dillen LR, Steger-May K, Sahrmann S, Schootman M (2016). Movement-pattern training to improve function in people with chronic hip joint pain: a feasibility randomized clinical trial. J Orthop Sports Phys Ther.

[34] Smeatham A, Powell R, Moore S, Chauhan R, Wilson M (2017). Does treatment by a specialist physiotherapist change pain and function in young adults with symptoms from femoroacetabular impingement? A pilot project for a randomised controlled trial. Physiotherapy.

[35] Wright AA, Hegedus EJ, Taylor JB, Dischiavi SL, Stubbs AJ (2016). Non-operative management of femoroacetabular impingement: a prospective, randomized controlled clinical trial pilot study. J Sci Med Sport.

[36] Hoit G, Whelan DB, Dwyer T, Ajrawat P, Chahal J (2020). Physiotherapy as an initial treatment option for femoroacetabular impingement: a systematic review of the literature and meta-analysis of 5 randomized controlled trials. Am J Sports Med.

[37] Wall PD, Dickenson EJ, Robinson D, Hughes I, Realpe A, Hobson R (2016). Personalised hip therapy: development of a non-operative protocol to treat femoroacetabular impingement syndrome in the FASHIoN randomised controlled trial. Br J Sports Med.

[38] Zhang C, Li L, Forster BB, Kopec JA, Ratzlaff C, Halai L (2015). Femoroacetabular impingement and osteoarthritis of the hip. Can Fam Physician.

[39] Kemp JL, King MG, Barton C, Schache AG, Thorborg K, Roos EM (2019). Is exercise therapy for femoroacetabular impingement in or out of FASHIoN? We need to talk about current best practice for the non-surgical management of FAI syndrome. Br J Sports Med.

[40] Gatz M, Driessen A, Eschweiler J, Tingart M, Migliorini F (2020). Arthroscopic surgery versus physiotherapy for femoroacetabular impingement: a meta-analysis study. Eur J Orthop Surg Traumatol.

[41] Aoyama M, Ohnishi Y, Utsunomiya H, Kanezaki S, Takeuchi H, Watanuki M (2019). A prospective, randomized, controlled trial comparing conservative treatment with trunk stabilization exercise to standard hip muscle exercise for treating femoroacetabular impingement: a pilot study. Clin J Sport Med.

[42] Casartelli NC, Bizzini M, Maffiuletti NA, Sutter R, Pfirrmann CW, Leunig M (2019). Exercise therapy for the management of femoroacetabular impingement syndrome: preliminary results of clinical responsiveness. Arthritis Care Res (Hoboken).

[43] Kemp JL, Coburn SL, Jones DM, Crossley KM (2018). The physiotherapy for femoroacetabular impingement rehabilitation study (physioFIRST): a pilot randomized controlled trial. J Orthop Sports Phys Ther.

[44] Monn S, Maffiuletti NA, Bizzini M, Sutter R, Naal FD, Leunig M (2022). Mid-term outcomes of exercise therapy for the non-surgical management of femoroacetabular impingement syndrome: are short-term effects persisting?. Phys Ther Sport.

[45] Kapron AL, Anderson AE, Aoki SK, Phillips LG, Petron DJ, Toth R (2011). Radiographic prevalence of femoroacetabular impingement in collegiate football players: AAOS exhibit selection. J Bone Joint Surg Am..

[46] Abate M, Scuccimarra T, Vanni D, Pantalone A, Salini V (2014). Femoroacetabular impingement: is hyaluronic acid effective?. Knee Surg Sports Traumatol Arthrosc.

[47] Khan W, Khan M, Alradwan H, Williams R, Simunovic N, Ayeni OR (2015). Utility of intra-articular hip injections for femoroacetabular impingement: a systematic review. Orthop J Sports Med.

[48] Lee YK, Lee GY, Lee JW, Lee E, Kang HS (2016). Intra-articular injections in patients with femoroacetabular impingement: a prospective, randomized, double-blind, cross-over study. J Korean Med Sci.

[49] Krych AJ, Griffith TB, Hudgens JL, Kuzma SA, Sierra RJ, Levy BA (2014). Limited therapeutic benefits of intra-articular cortisone injection for patients with femoro-acetabular impingement and labral tear. Knee Surg Sports Traumatol Arthrosc.

[50] Park JS, Jang YE, Nahm FS, Lee PB, Choi EJ (2013). Efficacy of intra-articular steroid injection in patients with femoroacetabular impingement. Korean J Pain.

[51] Ebert JR, Raymond AC, Aujla RS, D'Alessandro P (2023). The effect of a formal nonoperative management program combining a hip injection with structured adjunctive exercise rehabilitation in patients with symptomatic femoroacetabular impingement syndrome. Am J Sports Med.

[52] Sánchez M, Guadilla J, Fiz N, Andia I (2012). Ultrasound-guided platelet-rich plasma injections for the treatment of osteoarthritis of the hip. Rheumatology (Oxford).

[53] Ibrahim V, Dowling H (2012). Platelet-rich plasma as a nonsurgical treatment option for osteonecrosis. PM R.

[54] De Luigi AJ, Blatz D, Karam C, Gustin Z, Gordon AH (2019). Use of platelet-rich plasma for the treatment of acetabular labral tear of the hip: a pilot study. Am J Phys Med Rehabil.

[55] Belk JW, Kraeutler MJ, Houck DA, Goodrich JA, Dragoo JL, McCarty EC (2021). Platelet-rich plasma versus hyaluronic acid for knee osteoarthritis: a systematic review and meta-analysis of randomized controlled trials. Am J Sports Med.

[56] Zhao J, Huang H, Liang G, Zeng LF, Yang W, Liu J (2020). Effects and safety of the combination of platelet-rich plasma (PRP) and hyaluronic acid (HA) in the treatment of knee osteoarthritis: a systematic review and meta-analysis. BMC Musculoskelet Disord.

[57] Tang JZ, Nie MJ, Zhao JZ, Zhang GC, Zhang Q, Wang B (2020). Platelet-rich plasma versus hyaluronic acid in the treatment of knee osteoarthritis: a meta-analysis. J Orthop Surg Res.

[58] Anzillotti G, Iacomella A, Grancagnolo M, Bertolino EM, Marcacci M, Sconza C et al. Conservative vs. surgical management for femoro-acetabular impingement: a systematic review of clinical evidence. J Clin Med. 2022;11(19). 10.3390/jcm11195852. 10.3390/jcm11195852 PMC9572846 36233719

[59] Mahmoud SSS, Takla A, Meyer D, Griffin D, O'Donnell J (2022). Arthroscopic hip surgery offers better early patient-reported outcome measures than targeted physiotherapy programs for the treatment of femoroacetabular impingement syndrome: a systematic review and meta-analysis of randomized controlled trials. J Hip Preserv Surg.

[60] Griffin DR, Dickenson EJ, Wall PDH, Achana F, Donovan JL, Griffin J (2018). Hip arthroscopy versus best conservative care for the treatment of femoroacetabular impingement syndrome (UK FASHIoN): a multicentre randomised controlled trial. Lancet.

[61] Realpe AX, Foster NE, Dickenson EJ, Jepson M, Griffin DR, Donovan JL (2021). Patient experiences of receiving arthroscopic surgery or personalised hip therapy for femoroacetabular impingement in the context of the UK fashion study: a qualitative study. Trials.

[62] Palmer AJR, Ayyar Gupta V, Fernquest S, Rombach I, Dutton SJ, Mansour R (2019). Arthroscopic hip surgery compared with physiotherapy and activity modification for the treatment of symptomatic femoroacetabular impingement: multicentre randomised controlled trial. BMJ.

[63] Hunter DJ, Eyles J, Murphy NJ, Spiers L, Burns A, Davidson E (2021). Multi-centre randomised controlled trial comparing arthroscopic hip surgery to physiotherapist-led care for femoroacetabular impingement (FAI) syndrome on hip cartilage metabolism: the Australian FASHIoN trial. BMC Musculoskelet Disord.

[64] Mansell NS, Rhon DI, Meyer J, Slevin JM, Marchant BG (2018). Arthroscopic surgery or physical therapy for patients with femoroacetabular impingement syndrome: a randomized controlled trial with 2-year follow-up. Am J Sports Med.

[65] Casartelli NC, Valenzuela PL, Maffiuletti NA, Leunig M (2021). Effectiveness of hip arthroscopy on treatment of femoroacetabular impingement syndrome: a meta-analysis of randomized controlled trials. Arthritis Care Res (Hoboken).

[66] Domb BG, Gui C, Lodhia P (2015). How much arthritis is too much for hip arthroscopy: a systematic review. Arthroscopy.

[67] Kemp JL, MacDonald D, Collins NJ, Hatton AL, Crossley KM (2015). Hip arthroscopy in the setting of hip osteoarthritis: systematic review of outcomes and progression to hip arthroplasty. Clin Orthop Relat Res.

[68] Rhon DI, Greenlee TA, Sissel CD, Reiman MP (2019). The two-year incidence of hip osteoarthritis after arthroscopic hip surgery for femoroacetabular impingement syndrome. BMC Musculoskelet Disord.

[69] Collins JA, Ward JP, Youm T (2014). Is prophylactic surgery for femoroacetabular impingement indicated? A systematic review. Am J Sports Med.

[70] Sogbein OA, Shah A, Kay J, Memon M, Simunovic N, Belzile EL (2019). Predictors of outcomes after hip arthroscopic surgery for femoroacetabular impingement: a systematic review. Orthop J Sports Med.

[71] Haefeli PC, Albers CE, Steppacher SD, Tannast M, Büchler L (2017). What are the risk factors for revision surgery after hip arthroscopy for femoroacetabular impingement at 7-year followup?. Clin Orthop Relat Res.

[72] Thomas GE, Palmer AJ, Batra RN, Kiran A, Hart D, Spector T (2014). Subclinical deformities of the hip are significant predictors of radiographic osteoarthritis and joint replacement in women. A 20 year longitudinal cohort study. Osteoarthritis Cartilage.

[73] Agricola R, Heijboer MP, Bierma-Zeinstra SM, Verhaar JA, Weinans H, Waarsing JH (2013). Cam impingement causes osteoarthritis of the hip: a nationwide prospective cohort study (CHECK). Ann Rheum Dis.

[74] Migliorini F, Maffulli N (2021). Arthroscopic management of femoroacetabular impingement in adolescents: a systematic review. Am J Sports Med.

[75] Fukase N, Murata Y, Pierpoint LA, Soares RW, Arner JW, Ruzbarsky JJ (2022). Outcomes and survivorship at a median of 8.9 years following hip arthroscopy in adolescents with femoroacetabular impingement: a matched comparative study with adults. J Bone Joint Surg Am..

[76] Lin LJ, Akpinar B, Bloom DA, Youm T (2021). Age and outcomes in hip arthroscopy for femoroacetabular impingement: a comparison across 3 age groups. Am J Sports Med.

[77] Frank RM, Lee S, Bush-Joseph CA, Salata MJ, Mather RC, Nho SJ (2016). Outcomes for hip arthroscopy according to sex and age: a comparative matched-group analysis. J Bone Joint Surg Am.

[78] Beck EC, Drager J, Nwachukwu BU, Jan K, Rasio J, Nho SJ (2021). Gender and age-specific differences observed in rates of achieving meaningful clinical outcomes 5-years after hip arthroscopy for femoroacetabular impingement syndrome. Arthroscopy.

[79] Saadat E, Martin SD, Thornhill TS, Brownlee SA, Losina E, Katz JN (2014). Factors associated with the failure of surgical treatment for femoroacetabular impingement: review of the literature. Am J Sports Med.

[80] Horner NS, Ekhtiari S, Simunovic N, Safran MR, Philippon MJ, Ayeni OR (2017). Hip arthroscopy in patients age 40 or older: a systematic review. Arthroscopy.

[81] Dijkstra P, Glyn-Jones S, Palmer A. Femoroacetabular impingement syndrome. UptoDate. 2023. Retrieved March 8, 2023, from https://www-uptodate-com.ucsf.idm.oclc.org/contents/femoroacetabular-impingement-syndrome.

[82] Kunze KN, Beck EC, Nwachukwu BU, Ahn J, Nho SJ (2019). Early Hip Arthroscopy for femoroacetabular impingement syndrome provides superior outcomes when compared with delaying surgical treatment beyond 6 months. Am J Sports Med.

[83] Davey MS, Hurley ET, Davey MG, Fried JW, Hughes AJ, Youm T (2022). Criteria for return to play after hip arthroscopy in the treatment of femoroacetabular impingement: a systematic review. Am J Sports Med.

[84] Reiman MP, Peters S, Sylvain J, Hagymasi S, Mather RC, Goode AP (2018). Femoroacetabular impingement surgery allows 74% of athletes to return to the same competitive level of sports participation but their level of performance remains unreported: a systematic review with meta-analysis. Br J Sports Med.

[85] Minkara AA, Westermann RW, Rosneck J, Lynch TS (2019). Systematic review and meta-analysis of outcomes after hip arthroscopy in femoroacetabular impingement. Am J Sports Med.

